# MuSyC dosing of adjuvanted cancer vaccines optimizes antitumor responses

**DOI:** 10.3389/fimmu.2022.936129

**Published:** 2022-08-19

**Authors:** David Taylor, Christian T. Meyer, Diana Graves, Rupashree Sen, Juan Fu, Emily Tran, Bilal Mirza, Gabriel Rodriguez, Cara Lang, Hanwen Feng, Vito Quaranta, John T. Wilson, Young J. Kim, Michael J. Korrer

**Affiliations:** ^1^ Department of Cancer Biology, Vanderbilt University Medical Center, Nashville, TN, United States; ^2^ Department of Molecular, Cellular and Developmental Biology, University of Colorado Boulder, Boulder, CO, United States; ^3^ Department of Pathology, Microbiology, and Immunology, Vanderbilt University Medical Center, Nashville, TN, United States; ^4^ Department of Oncology, Johns Hopkins University School of Medicine, Baltimore, MD, United States; ^5^ College Arts and Sciences, Vanderbilt University, Nashville, TN, United States; ^6^ Department of Surgery, Vanderbilt University Medical Center, Nashville, TN, United States; ^7^ Department of Biochemistry, Vanderbilt University, Nashville, TN, United States; ^8^ Department of Chemical and Biomolecular Engineering, Vanderbilt University, Nashville, TN, United States; ^9^ Oncology Chair, Global Development, Regeneron Pharmaceuticals, Inc., Tarrytown, NY, United States; ^10^ Department of Otolaryngology Head and Neck Surgery, Vanderbilt University Medical Center, Nashville, TN, United States

**Keywords:** Multidimensional Synergy of Combinations (MuSyC), TLR7/8 agonist, STING agonists, cancer vaccine, antitumor/cytotoxic activity, T-cell priming, antigen presenting cell, activation markers

## Abstract

With the clinical approval of T-cell–dependent immune checkpoint inhibitors for many cancers, therapeutic cancer vaccines have re-emerged as a promising immunotherapy. Cancer vaccines require the addition of immunostimulatory adjuvants to increase vaccine immunogenicity, and increasingly multiple adjuvants are used in combination to bolster further and shape cellular immunity to tumor antigens. However, rigorous quantification of adjuvants’ synergistic interactions is challenging due to partial redundancy in costimulatory molecules and cytokine production, leading to the common assumption that combining both adjuvants at the maximum tolerated dose results in optimal efficacy. Herein, we examine this maximum dose assumption and find combinations of these doses are suboptimal. Instead, we optimized dendritic cell activation by extending the Multidimensional Synergy of Combinations (MuSyC) framework that measures the synergy of efficacy and potency between two vaccine adjuvants. Initially, we performed a preliminary *in vitro* screening of clinically translatable adjuvant receptor targets (TLR, STING, NLL, and RIG-I). We determined that STING agonist (CDN) plus TLR4 agonist (MPL-A) or TLR7/8 agonist (R848) as the best pairwise combinations for dendritic cell activation. In addition, we found that the combination of R848 and CDN is synergistically efficacious and potent in activating both murine and human antigen-presenting cells (APCs) *in vitro*. These two selected adjuvants were then used to estimate a MuSyC-dose optimized for *in vivo* T-cell priming using ovalbumin-based peptide vaccines. Finally, using B16 melanoma and MOC1 head and neck cancer models, MuSyC-dose–based adjuvating of cancer vaccines improved the antitumor response, increased tumor-infiltrating lymphocytes, and induced novel myeloid tumor infiltration changes. Further, the MuSyC-dose–based adjuvants approach did not cause additional weight changes or increased plasma cytokine levels compared to CDN alone. Collectively, our findings offer a proof of principle that our MuSyC-extended approach can be used to optimize cancer vaccine formulations for immunotherapy.

## Introduction

U.S. Food and Drug Administration (FDA) approval of T-cell–dependent immune checkpoint inhibitors (ICIs) in multiple tumors has re-engaged translational strategies to increase tumor-specific T cells’ frequency, diversity, and/or function (1–3). Consensus evidence supports this rationale because high T-cell infiltration in the tumor microenvironment often correlates with clinical response to approved anti Programmed cell death protein 1 (αPD1) and anti Programmed death-ligand 1 (αPDL1) blocking antibodies (4, 5). Hence, one promising approach to promote ICI’s clinical efficacy is developing therapeutic cancer vaccines that can generate an increased tumor-specific T-cell response (6–8). However, previous clinical trials of cancer vaccines as monotherapies have suggested these early generations of cancer vaccines cause only a modest tumor infiltration of tumor-specific T cells (9–11). While the limited efficacy of cancer vaccines in cancer patients has been attributed to several mechanisms, one limitation of vaccine trials may stem from suboptimal antigen-presenting cell (APC) stimulation and potentially toxic inflammatory side effects of the adjuvants.

Several signaling pathways sense adjuvants for APC stimulation, namely, NOD-like receptors (NLRs), RIG-I–like receptors (RLRs), Toll-like receptors (TLRs), and the Stimulator of Interferon Genes (STING) receptor. Adjuvant agonism of these targets leads to the upregulation of costimulatory molecules (i.e., CD80, CD86, and CD40) and major histocompatibility complexes (i.e., MHCI and MHCII) to promote T-cell priming and activation (12–15). In general, single adjuvanted cancer vaccines have failed to produce a sufficient tumor-specific response against cancer, prompting exploration of adjuvant combinations as a strategy to strengthen antitumor T-cell responses (16–19). Preclinical studies from different classes of TLR agonists have shown that their combination could increase the antitumor response compared to the single agents (20–23). However, combining a MyD88-dependent adjuvant with another MyD88-dependent adjuvant (24, 25), or utilizing more than two adjuvants, has led to modest antineoplastic responses (21), making the combinatorial choice imperative for optimizing responses. Altogether, the primary shortcoming of these studies is that they are efficacy-focused without consideration of potency (**Table 1**) and toxicity. Clinically, combining adjuvants without optimization, for instance, using a high dose of both adjuvants, leads to an increase in toxicities (26, 27). In short, these empirical approaches have not adopted a rigorous quantitative definition of the synergy of adjuvants.

**Table 1:** Key definitions.

Potency	The amount of drug required to produce an effect. Generally, it is quantified by measuring the EC_50_, the concentration or dose of drug that causes 50% of maximum effect. The lower the EC_50_, the more potent the drug.
Efficacy	The maximum effect that a drug can produce regardless of dose. Classically, efficacy is quantified by the maximal effect (e_max_).
Synergistic potency	Increase in the potency (decrease in EC_50_), owing to the presence of another drug.
Synergistic efficacy	Increase in e_max_ with the combination compared to the most efficacious single agent.

Optimal dosing of adjuvants importantly addresses the critical concept of “off-target” and adverse effects in patients. For example, MyD88 and Nuclear factor kappa B (NF-κB) signaling in the tumor through TLR stimulation has been shown to have oncogenic potential (28–32). In addition, adjuvants have been demonstrated to induce the systemic release of Tumor necrosis factor (TNF), Interleukin 6 (IL-6,) Interleukin-1 (IL-1), and Macrophage Inflammatory Protein-1 Alpha (MIP1-α), which can mediate cytokine storm and limit clinical combination strategies (24, 33). Therefore, the development of adjuvanted cancer vaccines must minimize some of these “off-target” consequences through appropriate dosing while maximizing T-cell tumor infiltration. Toward this goal, we extended our recently described synergy framework, the Multidimensional Synergy of Combinations (MuSyC), for the adjuvant combination (34–36). MuSyC distinguishes between two types of synergy: synergy of efficacy, which quantifies the maximal effect, and synergy of potency, which measures potency change due to the combination (34–36). Furthermore, the MuSyC framework removes the inherent biases and ambiguities of the two most common drug synergy principles, Loewe’s Dose Equivalence Principle and the Multiplicative Survival Principle introduced by Bliss (36). The MuSyC algorithm unifies these two principles to make a consensus framework for quantifying drug combination synergy. Therefore, we hypothesized that utilizing the MuSyC algorithm to guide how adjuvants are combined will maximize therapeutic efficacy while minimizing the total dose and reducing off-target effects in cancer vaccines

## Results

### The MuSyC algorithm measures the synergy of STING and TLR agonists

To determine the optimal adjuvant combination for activating APCs, we screened the following major PRR class adjuvants known to activate murine bone marrow–derived dendritic cells (mBMDCs): STING agonist cyclic dinucleotides (CDN), TLR4 agonist monophosphoryl lipid A (MPL-A), TLR7/8 agonist resiquimod (R848), TLR9 agonist CpG oligonucleotide (CpG), NLR agonist tri-DAP (NLL), and RLR agonist 5’ppp-dsRNA (RLL) (13, 15, 37, 38). After identifying saturating doses for CD86 for each adjuvant, we identified CDN as the most efficacious single agent for inducing MHCI, CD86, and TNF (signal 1, signal 2, and signal 3, respectively) on mBMDCs (**Supplementary Figure 1**). With the saturating dose of CDN, we combined it with the saturating amount of the other adjuvants, which we termed “Max-dose”. At Max-dose for the combinations, CDN significantly enhances all three signals compared to the single agents alone (**Supplementary Figure 2**). However, NLL, RLL, and CpG Max-dose combinations significantly antagonized MHCI, CD40, and TNF than CDN alone (**Supplementary Figure 2**). Only Max-dose combinations of R848 or MPL-A significantly improved at least one of the signals compared to CDN alone (**Supplementary Figure 2**). Therefore, we chose MPL-A or R848 as potential choices to combine with CDN.

**Figure 1:**
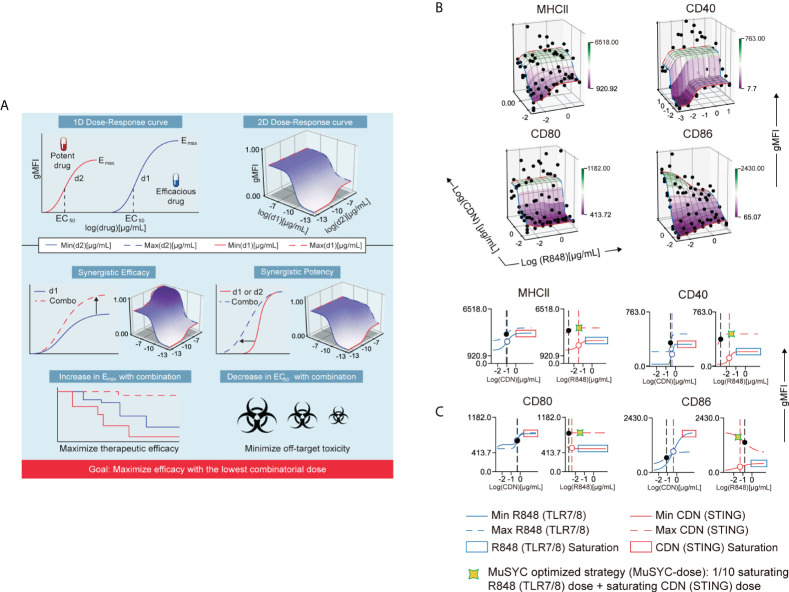
The combination of CDN and R848 is synergistically efficacious and potent. **(A)** MuSyC algorithm classification scheme. **(B)** MuSyC algorithm-generated drug synergy diagrams for STING and TLR7/8 agonists activation of bone marrow–derived dendritic cells (mBMDCs). The y-axis is the log concentration of CDN (STING), the x-axis is the log concentration of R848 (TLR7/8), and the z-axis is the geometric mean fluorescence intensity (gMFI) of multiple activation markers. Points are experimentally measured conditions. The surface is the fit to the MuSyC equation, which quantifies the synergistic potency and efficacy. The solid red line R848 single-agent dose-response. The solid blue line represents CDN single-agent dose-response. The blue dashed line is the max dose of R848 plus increasing amounts of CDN. The red dashed line represents the max dose of CDN plus increasing doses of R848. **(C)** One-dimensional graphs displaying an open circle for the EC_50_ for the single agents and a solid black circle for the new EC_50_ in the presence of the combinatorial agent. The vertical dashed lines represent the EC_50_ of the respective curve. Optimization and derivation of the MuSyC-dose for the combination (1/10th saturating dose R848 and saturating dose CDN).

The STING-TLR titrations using geometric mean fluorescence intensity (gMFI) of CD86, MHCII, CD80, and CD40 signals were analyzed by the MuSyC algorithm to generate three-dimensional drug synergy diagrams to assess synergistic potency and efficacy (**Table 1** and **Figure 1A**). The results show no change in the MPL-A and CDN combination for the maximal activation (e_max_) of MHCII, CD40, CD86, and CD80 markers compared to CDN alone, the most efficacious single agent (**Supplementary Figures 3B, C**). However, CDN increased its potency (left shift in the EC_50_) in the presence of MPL-A for CD40 and CD80, and MPL-A increased its potency in the presence of CDN for MHCII (**Supplementary Figures 3B, C**). Therefore, the combination of CDN and MPL-A is considered synergistically potent in the MuSyC framework.

In contrast, the R848 and CDN combination increased the e_max_ compared to CDN alone for MHCII, CD40, and CD80 (**Figure 1B**). In addition, R848 increased its potency in the presence of CDN for the same markers (**Figure 1B**). Hence, this combination is synergistically potent and efficacious for mBMDC activation (**Figure 1B**). However, R848 plus CDN, as with the MPL-A combination, antagonized costimulatory molecule CD86, revealing an antagonistic effect with the combination that may affect the priming of T cells (**Supplementary Figure 3B** and **Figure 1B**). To offset this “antagonistic” effect, we derived a MuSyC synergy dosing strategy (MuSyC-dose) for the CDN and R848 combination, where we use the saturating dose from CDN and 1/10th of the saturating amount of R848 (**Figure 1C**). The MuSyC-dose strategy could potentially rescue the antagonistic effect on CD86 activation while maintaining a similar e_max_ for the other markers. Therefore, the MuSyC algorithm can enable the combination dosing strategy that simultaneously maximizes multiple costimulatory molecules’ expression while minimizing the amount of adjuvant necessary.

### The MuSyC-dose can optimize activation for multiple APCs *in vitro*

**Figure 2:**
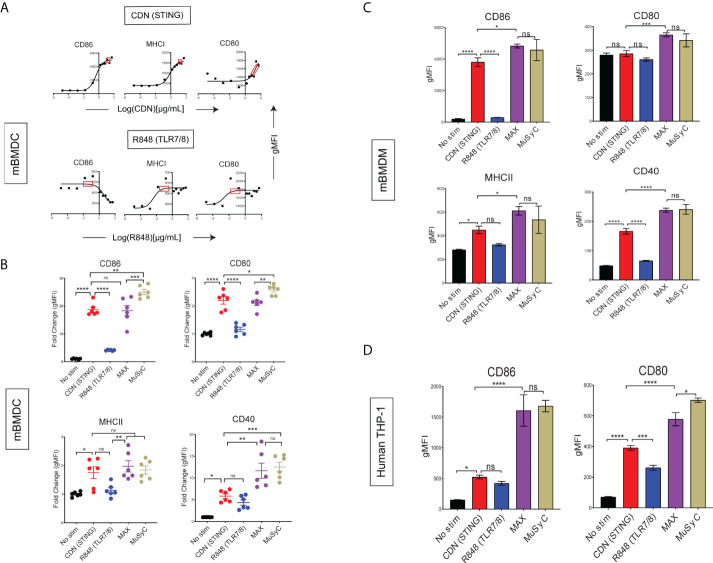
MuSyC-dose can optimize activation for multiple APCs *in vitro.* **(A)** Corresponding dose-response curves for CDN (STING) and R848 (TLR7/8) for the activation of mBMDCs with the saturation range for each adjuvant in the red box. 20 µg/ml was chosen for CDN and 0.1 µg/ml was chosen for R848. **(B)** mBMDCs activated with R848, CDN, Max-dose (CDN of 20 µg/ml + R848 of 0.1 µg/ml), and MuSyC-dose (20 µg/ml + R848 of 0.01 µg/ml). The fold change gMFI normalized to the average no stim control of the activation markers are shown. **(C)** Murine bone marrow–derived macrophages (mBMDM) activation with the doses selected through the corresponding dose-response (Supplementary Figure 4A). **(D)** Human monocytic cell line (TH-P1) activation with appropriate doses (Supplementary Figure 4B). Figure 2B is given in mean ± SEM for two independent experiments. All other data are given in mean ± S.D. of three technical replicates. *P < 0.05, **P < 0.01, ***P < 0.001, and ****P < 0.0001; one-way analysis of variance (ANOVA) for multiple comparisons. ns, not significant.

To test and validate that the MuSyC-dose strategy (saturating CDN plus 1/10th saturating R848) maximizes e_max_ and counteracts potential antagonistic effects, we utilized the same mBMDC activation model. We first performed dose-response to determine the saturating quantity for CDN and R848 (**Figure 2A**). The saturating dose for CDN was 20 and 0.1 µg/ml for R848 and, when combined, is the Max-dose. On the other hand, the MuSyC-dose approach used the saturating dose of CDN (20 µg/ml) and 1/10th saturating dose for R848 (0.01 µg/ml). We then compared Max-dose and MuSyC-dose in an mBMDC activation assay to confirm whether the combination is synergistically efficacious and potent. We found that the Max-dose group had synergistic efficacy, as demonstrated by enhanced average expression of MHCI, MHCII, and CD40 on mBMDCs compared to CDN (STING) alone (**Figure 2B**). The MuSyC-dose maintained or significantly increased that synergistic efficacy at a lower total combinatorial dose, confirming the synergistically efficacious and potent effects of the MuSyC-dose strategy for mBMDCs (**Figure 2B**). We also saw a slight decrease in average expression for Max-dose compared to CDN for CD86 and a rescue effect with MuSyC-dose, which we predicted the MuSyC-dose would counteract (**Figure 2B**). Hence, we can validate the MuSyC-dose without performing the checkerboard method with MuSyC analysis (50 samples) and instead utilize single-agent dose responses with the MuSyC-dose strategy (10 samples).

Next, we wanted to determine whether the MuSyC-dose would translate to other types of APCs. Therefore, we established a dose-response for R848 and CDN on murine bone marrow–derived macrophages (mBMDMs) (**Supplementary Figure 4A**) and human monocytic cell line (THP-1) (**Supplementary Figure 4B**) and identified the saturating dose for each agent per model. We then compared the Max-dose and MuSyC-dose (CDN-max R848, 1/10th max) for mBMDM (**Figure 2C**) and human THP-1 cells (**Figure 2D**). We showed that MuSyC dosing potentially enhanced stimulatory effects compared to CDN alone, and similar or better activation effects relative to the Max-dose combination in THP-1 cells and mBMDMs. These data suggest that the MuSyC-dose strategy developed using mBMDC applies to both mBMDM and human THP-1 cells.

### CDN-based vaccines induce optimal T- cell priming *in vivo*

**Figure 3:**
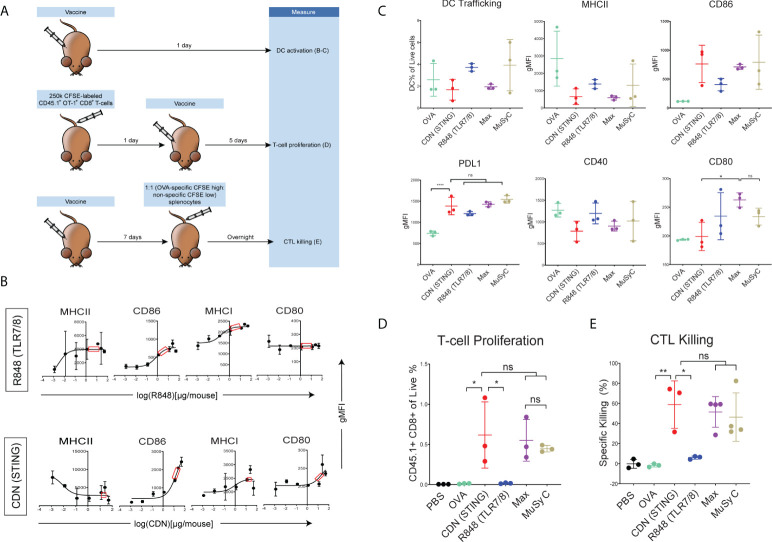
CDN-based vaccines induce optimal T- cell priming. **(A)** *In vivo* T-cell priming schematic. **(B)** Female C57BL/6 mice (n = 2) were subcutaneously injected with 20μg of full-length ovalbumin protein vaccine with increasing doses of R848 (TLR7/8) and CDN (STING). CD11c + MHCII+ DC activation status is shown. Saturating doses of 2 µg was chosen for R848 and 20 µg for CDN for all remaining *in vivo* experiments. **(C)** Female C57BL/6 mice (n = 3) were injected with OVA alone or OVA plus R848, CDN, Max-dose (R848 of 2 µg per mouse + CDN of 20 µg per mouse), and MuSyC-dose (R848 of 0.2 µg per mouse + CDN of 20 µg per mouse). The gMFI for activation/inhibitory receptors (CD80, CD86, PDL1, MHCII, and CD40) and the percentage of murine DCs of total live cells in the lymph node were measured. **(D)** A total of 250,000 CFSE-labeled CD45.1+ OT-1 CD8 T cells were intravenously injected into female C57BL/6 mice (n = 3). Mice were injected with the corresponding vaccine. The percentage of CD45.1+ of the live cells is shown. **(E)** C57BL/6 mice (n = 3–4) were subcutaneously injected with PBS or the appropriate vaccine. CFSE high OVA peptide-pulsed:CFSE low splenocytes (1:1) were intravenously injected. The specific killing percentage was measured with the equation shown [ratio = low peak (non-specific) / high peak (OVA-specific). Specific killing = 1 − (PBS ratio average)/(exp. ratio) * 100]. All data are given in mean ± S.D. *P < 0.05, **P < 0.01, ***P < 0.001, and ****P < 0.0001; one-way analysis of variance (ANOVA) for multiple comparisons. ns, not significant.

After demonstrating that MuSyC generated combination dose (MuSyC-dose) of CDN and R848 was equivalent/comparable to the Max-dose across multiple APC models *in vitro*, we tested whether the MuSyC-dose also leads to synergistic T-cell activation *in vivo* (**Figure 3A**). First, we established the saturating doses of the adjuvants R848 and CDN *in vivo* using the maturation of CD11c+ MHCII+ dendritic cells (**Supplementary Figure 5**) in the draining lymph node with CDN- and R848-adjuvanted ovalbumin-based peptide vaccines (OVA) (**Figure 3B**). We identified 2 µg per mouse for R848 and 20 µg per mouse for CDN (**Figure 3B**) as the saturating dose based on the expression of activation markers MHCII CD86, MHCI, and CD80 on draining lymph node DCs.

Comparing MuSyC-dose to Max-dose and CDN alone resulted in similar DC activation for surrogate markers MHCII, CD86, PDL1, and CD40 (**Figure 3C**). R848 alone, in general, had only a modest impact on the activation of the combinations (**Figure 3C**). Thus, the activation status for the combinations is primarily CDN-driven. Finally, we analyzed the MuSyC dosing strategy to optimize T-cell priming *in vivo*. Adoptive transfer of Carboxyfluorescein succinimidyl ester CFSE-labeled OVA-specific T cells followed by vaccination and 5-day incubation determined that CDN and both combinations led to at least 95% of CD45.1 OT-1 T cells proliferating (**Supplementary Figure 6**), essentially saturating that effect. Furthermore, there was no significant difference between CDN and the combinations in the CD45.1+ CD8+ percentage of the live splenocytes (**Figure 3D**). On the other hand, the R848-adjuvanted peptide vaccine did not increase the CD45.1+CD8+ rate compared to OVA alone. Again, the response of the combinations seems to be CDN-based. Next, we tested the effect of the proliferative response on the antigen-specific killing by vaccinating the mice and intravenously injecting CFSE-high labeled OVA-specific splenocytes and CFSE-low non-specific splenocytes. Sixteen hours post-vaccination, the splenocytes were extracted, and antigen-specific killing was calculated. Yet again, as with the T-cell proliferation and APC activation, there was no significant difference between the CDN-adjuvanted peptide vaccine and the combinations in cytolytic activity (**Figure 3E**). In addition, the R848-based vaccine did not significantly induce killing compared to the vaccine alone. Collectively, the CDN saturates the T-cell priming effect at the doses tested.

### MuSyC-dose optimizes the antitumor response *in vivo* by modifying the tumor microenvironment

**Figure 4:**
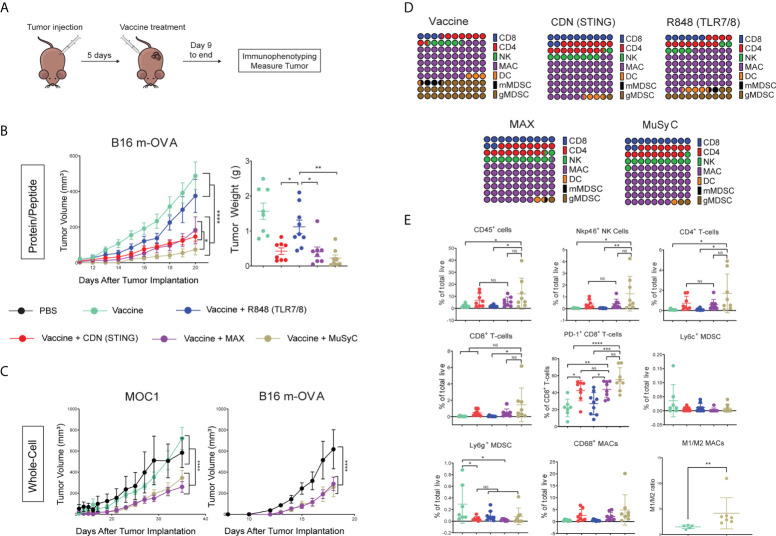
MuSyC dosing optimizes the antitumor response *in vivo* through modulating the tumor microenvironment. **(A)** General vaccination strategy for tumor models **(B)** 10^5^ B16 m-OVA tumor cells were subcutaneously injected on one flank of C57BL/6 mice (n = 10–11). Mice were then subcutaneously injected with 20 μg of full-length ovalbumin protein vaccine alone, 20 µg of vaccine administered with 2 μg per mouse R848 (TLR7/8), 20 μg per mouse CDN (STING), Max-dose (R848 of 2 μg per mouse + CDN of 20 µg per mouse), or MuSyC-dose (R848 of 0.2 μg per mouse + CDN of 20 μg per mouse) in the opposite flank. B16 m-OVA tumor curve and final day tumor weights are shown. **(C)** 10^5^ B16 m-OVA and 2 × 10^6^ MOC1 were subcutaneously injected in one flank C57BL/6 mice (n = 8–12). Mice were subcutaneously injected with PBS, 10^6^ whole-cell vaccine GVAX (B16 m-OVA or MOC1-derived GVAX), and GVAX administered with Max-dose and MuSyC-dose. B16 and MOC1 tumor curves are shown. **(D)** Percentage of CD45 for the following cell types is shown for B16-moVA peptide vaccination model: NK cells (CD11b-Nkp46+), CD8+ T cells (CD11b-Nkp46-CD4-CD8+), CD4+ T cells (CD11b-Nkp46-CD8-CD4+), gMDSC (CD11b+MHCII-CD68-CD11c-Ly6G+), mMDSC(CD11b+MHCII-CD68-CD11c-Ly6C+), DC (CD68-CD11c+MHCII+), and MACs (CD11c- CD68+). **(E)** Percentage of total live of different cell types that include CD45+ immune cells, NK cells, CD4+ T cells, CD8+ T cells, PD-1+ CD8+ T cells, CD69+ CD8+ T cells, mMDSC, gMDSC, DCs, and MACs. **(F)** M1(CD206−)/M2(CD206+) ratio of CD68+ MACs. MOC1 whole-cell and B16 m-OVA peptide models are given in mean ± SEM with two independent experiments. All other data are given in mean ± S.D. of biological replicates. *P < 0.05, **P < 0.01, ***P < 0.001, and ****P < 0.0001; one-way or two-way analysis of variance (ANOVA) for multiple comparisons. NS, not significant.

Next, we tested the antitumor response of Max-dose and MuSyC-dose *in vivo*. We first compared Max and MuSyC adjuvant doses using a therapeutic vaccine model with B16 melanoma tumor cells expressing the model tumor antigen ovalbumin (B16-mOVA). Mice were injected with B16-mOVA tumors on the flank and, 5 days later, vaccinated on the opposite flank (**Figure 4A**). The peptide vaccine consisted of endotoxin-free ovalbumin as the antigen source with or without the adjuvants. As expected, vaccination with OVA alone did not reduce tumor volume, indicating adjuvants’ critical role in the antitumor immune response to vaccination. The addition of R848 to the OVA vaccine enhanced the antitumor response, but the effect seems to start to diminish 12 days post-treatment. In contrast, the CDN-based vaccine significantly decreased the tumor volume and is more durable than the R848 vaccine (**Figure 4B**). The Max-dose vaccine does not add any additional benefit to the CDN vaccine. However, utilizing 1/10th of R848, the MuSyC-dose significantly reduced the tumor volume than the other groups, including Max-dose and CDN alone vaccines (**Figure 4B**). This tumor reduction trended with the tumor weights, but the MuSyC-dose tumor weight was not significant compared to Max-dose and CDN (**Figure 4B**).

We also adopted the cell-based GVAX vaccine platform, irradiated cancer cells genetically modified to secrete granulocyte-macrophage colony-stimulating factor (GM-CSF) (39). We determined the antitumor effects of MuSyC-dose versus Max-dose adjuvanted GVAX using two murine tumor models, B16-mOVA melanoma, and the head and neck tumor MOC1. In both cases, MuSyC-dose or Max-dose significantly lowered the tumor volume compared to Phosphate-buffered saline PBS or GVAX alone (**Figure 4C**). However, there was no significant difference in the Max-dose and the MuSyC-dose responses (**Figure 4C**). Therefore, similar to our *in vitro* findings, utilizing a lower total dose of combinatorial R848 plus CDN (MuSyC-dose) either has better or similar reactions to Max-dose, validating the MuSyC algorithm.

Next, we wanted to identify immune cell types involved in the antitumor response. Therefore, we utilized the ovalbumin-based peptide vaccine with the B16 m-OVA tumor cell melanoma model. Twenty-one days post–tumor inoculation, tumors were extracted and immunophenotyped through flow cytometric analysis. We first examined the makeup of the CD45+ immune cell infiltrate, including CD8+ T cells, CD4+ T cells, natural killer (NK) cells, myeloid-derived suppressors cells (MDSCs), DCs, and macrophages (MACs) (**Supplementary Figure 7**). Results indicate various measured immune cell populations induced by vaccine alone, including approximately 30% of total MDSCs (**Figure 4D**). In addition, the R848 vaccine reduced the MDSC percentage of CD45+ by roughly half and doubled the CD8+ T cells compared to the vaccine alone (**Figure 4D**). CDN vaccine further decreased the rate of MDSCs and increased the CD8+ T cell, NK cell, and CD4+ T-cell percentages compared to the R848 vaccine alone (**Figure 4D**). Interestingly, the MuSyC-dose and Max-dose groups had over 55% macrophages, potentially meaning that the efficacious antitumor response was myeloid-derived (**Figure 4D**). However, there are no significant differences in the percentage of CD45+ immune populations between CDN, Max-dose, and MuSyC-dose vaccines (**Figure 4D**).

Finally, we measured the percentage of total live for the immune infiltrate. The vaccine alone treatment group led to an average of 2% of CD45+ immune cells in the tumor (**Figure 4E**). R848-adjuvanted vaccine had no impact on the infiltration of total CD45+ infiltrate (**Figure 4E**). However, the R848-based vaccine decreased the average total MDSC penetrate compared to the vaccine alone, which is a potential mechanism by which this treatment induced an antitumor response. Both CDN and Max-dose vaccines increased the average rate of CD45+ in the tumor by more than 2.5 times compared to R848 or vaccine alone (**Figure 4E**). Moreover, although not significantly, both groups enhanced the percentage of NK cells, CD8 T cells, CD4 T cells, and CD68+ MACs and decreased total MDSCs. The CD8+ T cells, specifically, have significantly higher CD69 and PD-1, making them more activated in CDN and Max-dose groups than the R848 and vaccine-only groups.

Interestingly, the MuSyC-dose treatment group had the highest average percentage of CD45+ immune cells in the tumor at approximately 12%, significantly higher than R848 and vaccine alone and roughly two times higher than CDN and Max-dose treatment groups (**Figure 4E**). These higher CD45+ immune infiltrates induced by the MuSyC-dose treatment led to an enhanced percentage of CD8+ T cells, CD4+ T cells, NK cells, and CD68+ MACs in the tumor compared to the other groups (**Figure 4E**). Of those CD68+ MACs, the average ratio of M1:M2 was approximately 4:1 with the MuSyC-dose vaccine, which is significantly higher than the non-adjuvanted vaccine (**Figure 4F**). (**Figure 4F**). In addition, MuSyC-dose, as with the CDN and Max-dose, had more activated CD8 T cells and lower total MDSC infiltrate to R848 and vaccine-only treatment groups (**Figure 4E**). Altogether, using MuSyC to guide combination dosing rationally, we achieved a tumor burden reduction that either outperforms or is similar to naively combining both drugs at the saturating dose.

### The MuSyC-dose vaccine induces no additional weight loss and decreases the plasma concentration of IL-6 compared to the CDN vaccine

**Figure 5:**
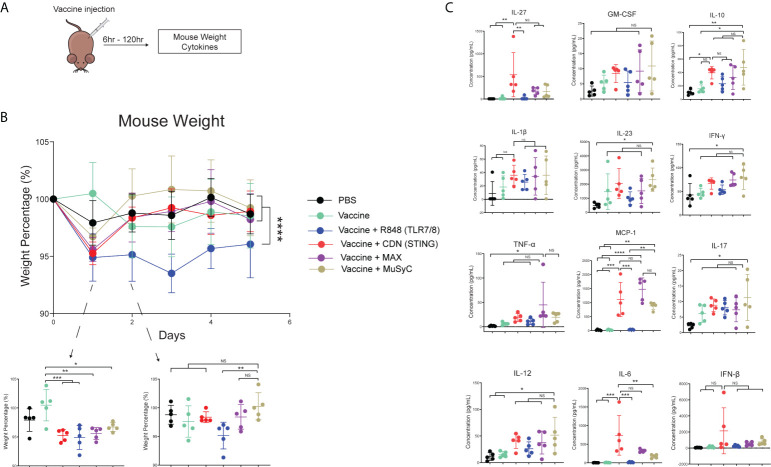
The MuSyC-dose vaccine induces no additional weight loss and decreases the plasma concentration of IL-6 compared to the CDN vaccine. **(A)** Vaccination schematic for mouse weight and cytokines **(B)** C57BL/6 mice (n = 5) was weighed initially and then were subcutaneously injected with PBS, 20 μg of full-length ovalbumin protein vaccine alone, 20 µg of vaccine administered with R848 of 2 μg per mouse (TLR7/8), CDN of 20 μg per mouse (STING), Max-dose (R848 of 2 μg per mouse + CDN of 20 µg per mouse), or MuSyC-dose (R848 of 0.2 μg per mouse + CDN of 20 μg per mouse) in one flank. Mouse weight was measured daily. **(C)** C57BL/6 mice (n = 5) were subcutaneously injected with the appropriate treatment. Six hours post-injection, mouse plasma was collected and measured for cytokines. *P < 0.05, **P < 0.01, ***P < 0.001, and ****P < 0.0001; one-way or two-way analysis of variance (ANOVA) for multiple comparisons. ns, not significant.

A vital component of the MuSyC algorithm is to reduce “off-target” effects through synergistically potent combinations. Therefore, we measured mouse weight and plasma cytokine levels to test potential adjuvanted vaccine-induced toxicities (**Figure 5A**). To measure vaccine-induced weight changes, mice were weighed for their initial weight (100%) and subsequently injected with PBS or the appropriate vaccine. Twenty-4 h post-injection, all adjuvanted vaccine mouse groups lost a significant amount of weight compared to the vaccine alone (**Figure 5B**). The weight loss was the least notable in the MuSyC-dose group compared to the vaccine alone (**Figure 5B**). The MuSyC-dose vaccinated mice have a more complete recovery than CDN and Max-dose groups compared to the R848-adjuvanted vaccine 48 h post-injection. However, the MuSyC-dose does not significantly affect weight loss compared to CDN or Max-dose vaccines for the 24- and 48-h timepoints. Interestingly, R848 single-agent vaccines induced persistent weight loss (**Figure 5B**). Moreover, CDN negates the combinations’ R848-induced ongoing weight loss effects, showing beneficial CDN-driven results. Overall, the combinations have no significant weight changes compared to CDN alone, suggesting no additional potential toxic effects.

Next, we evaluated plasma cytokine levels generated by adjuvanted vaccine treatment 6 h post-vaccination. The results showed that R848-adjuvanted vaccine does not significantly change plasma cytokine levels compared to PBS or vaccine alone (**Figure 5C**). Compared to PBS, vaccine alone, and R848 vaccine, CDN vaccine significantly increased IL-6, IL-27, and MCP-1 (CCL2). IL-6 is considerably lower in the MuSyC-dose treatment group than in the CDN group (**Figure 5C**). Notably, Max-dose or MuSyC-dose vaccines do not significantly affect the remaining cytokines to the CDN treatment. Therefore, the Max-dose or MuSyC-dose does not potentiate weight loss or plasma cytokines compared to CDN alone, unlike the common clinical trend where Max-dose combinations increase toxicities.

## Discussion

Here, we extended the MuSyC algorithm, previously used to classify targeted chemotherapeutic drug combinations, to guide dose selection when combining immune-activating agents. Our approach optimizes the adjuvant combinations to maximize efficacy and minimize total dose for *in vitro* and *in vivo* applications. The MuSyC algorithm classified the combination of CDN and R848 as synergistically efficacious and potent for APC activation. We then rationally calculated an optimal dose, termed the MuSyC-dose, by multiparametric optimization of immune activation across a matrix of dose combinations. Future work includes defining a Pareto front enabling the optimization of dosing selection algorithmically rather than heuristically; for example, by combining our approach with system biology models of the immune dynamics. This would enable optimizing combinatorial doses for CDN and TLR mixtures. Regardless, our derivation of the MuSyC-dose for the CDN and R848 induced similar or better APC activation effects compared to Max-dose *in vitro* for multiple models.

To the best of our knowledge, we are the first to utilize an algorithm *in vitro* to derive a synergy dosing strategy for immune-activating agents for *in vivo* experimentation. However, in the present study, our *in vitro* APC activation data did not recapitulate the APC activation effects *in vivo* for Max-dose or MuSyC-dose because of CDN stimulation’s overpowering impact, leading to the saturating effects on the clonal expansion of cytotoxic T cells. Notably, consistent with previous studies, the “free” R848-adjuvanted peptide vaccine did not lead to or provide any additional benefit to T-cell priming for the combinations (12, 40, 41). Multiple approaches to improve the T-cell responses of R848, CDN, or other TLR-based vaccines have been reported, which include the following: utilizing higher doses to allow the agent to remain in the body longer (42–44), formulating with emulsion agents to generate a slow-release “depot effect” (45, 46), and encapsulating with nanoparticles to improve delivery (40, 41, 47). Nevertheless, our present work and other studies have shown that R848 and CDN induce myeloid-based mechanisms, i.e., reduction of MDSCs (48, 49), to reduce tumor burden. Hence, APC activation and T-cell priming should not be the final determining factor on which adjuvant combinations will be successful at therapeutically curing tumors as CDN, MuSyC-dose, and MAX-dose had similar T-cell priming effects. Still, the MuSyC-dose vaccine increased the antitumor responses compared to CDN alone.

This disconnect emphasizes the challenges in translating between *in vitro* and *in vivo* dosing strategies. As stated earlier, we predicted the synergistic MuSyC-dose for *in vitro* APC activation would be similar for APC activation *in vivo* and would lead to synergistic T-cell priming and antitumor response. However, the MuSyC-dose only led to synergistic effects on the antineoplastic response, similar to when TLR agonist and GVAX are combined (50). Thus, tumor models are the most accurate way to gauge how a combination will work. For that reason, one could argue we should bypass the *in vitro* work, *in vivo* APC activation, and T-cell priming assays and perform tumor models with MuSyC analysis. The two apparent flaws to this approach are time (tumor processing and immunophenotyping) and cost (approximately 75 mice per tumor model and combination). Moreover, the MuSyC analysis and optimization strategy would be more complex due to the numerous parameters that would need to be included, such as final day tumor volume, T-cell infiltration, and MDSC percentage. Therefore, we currently point out that utilizing *in vitro* APC activation with MuSyC analysis/optimization and translating that to *in vivo* is a sufficient and cost-effective approach for combining adjuvants, as demonstrated with the *in vitro* derived MuSyC-dose, leading to a synergistic decrease in tumor volume and modulation of the tumor microenvironment.

Clinically, the importance of therapeutic cancer vaccines manipulating the host–tumor interaction has increasingly become evident for inducing clinical responses, considering that many tumors have immunosuppressive microenvironments (51–53). Both R848 and CDN have been used in clinical trials either as a single-agent intratumoral/topical treatment (54, 55) or as an adjuvant for vaccine formulations (56), but the combination has yet to be studied. Generally, CDN and R848 treatments are well tolerated clinically, and we show that the combination possibly will not potentiate the side effects. Clinical trials have utilized combinatorial adjuvants for cancer treatments, and various preclinical studies are identifying methods to optimize drug combinations. Here, we utilized the MuSyC algorithm to generate a ratio for the CDN + R848 as a strategy to optimize APC activation for T-cell priming. Although we have data showing this ratio works for human monocytes, dosing strategies will have to be identified in non-human primates for clinical trials. Here, we provide a proof of concept by which one can identify logical combinations and methods of optimization for those future trials.

The present study treated tumor-bearing mice with R848- and CDN-based vaccine treatments and performed immunophenotyping on the tumors 16 days post-treatment. Specifically, the MuSyC-dose–adjuvanted vaccines optimized the antitumor response and induced novel changes to the tumor microenvironment. Moreover, the MuSyC-dose vaccine significantly increased cytotoxic T cells in the tumor, and correlations have shown that increased CD8+ infiltrate is associated with a better response to anti–PD-1 therapy (5). The boost in CD8 T cells and enhanced PD-1 expression make MuSyC-dose–adjuvanted cancer vaccines a good candidate for combination with checkpoint blockade.

In addition to the increase in tumor-infiltrating lymphocytes, the MuSyC-dose vaccine CD45+ population contained a high percentage of macrophages, suggesting a myeloid-based mechanism for the antitumor response. Furthermore, CDN and R848 without antigen have been shown to cause an antineoplastic response, meaning responses in these studies were not due to the priming of antigen-specific T cells [40-43]. Overall, we point out that MuSyC-dose–based vaccines modulate the tumor microenvironment to optimize tumor response. In conclusion, our work questions the long-standing assumption regarding the superiority of using the maximum permissible concentrations when combining immunoadjuvants. Instead, by measuring multiple markers of immune activation, our work detailed a more nuanced view of adjuvant synergy, thereby paving the way for a more rigorous approach in deriving dosing strategies for vaccine adjuvants.

## Materials and methods

### Mice

Female C57BL/6 (strain #: 000664), female and male C57BL/6-Tg(TcraTcrb)1100Mjb/J (OT-1) (strain #: 003831), and female and male B6.SJL-Ptprca Pepcb/BoyJ (CD45.1) (strain #: 002014) mice were purchased from The Jackson Laboratory. OT-1 × CD45.1 and all other mice were housed according to the Vanderbilt University Medical Center Animal Care and Use Committee rules. All animal experiments were reviewed and approved by the Institutional Animal Care and Use Committee at Vanderbilt University Medical Center (M1900004-00). According to the National Institutes of Health (NIH) guidelines, the Animal Welfare Act, and the US federal law, all experiments were performed.

### Cell lines

B16 m-OVA melanoma, Mouse Oral Squamous Cell Carcinoma (MOC1), B78H1-GM-CSF, and human monocytic THP-1 were grown in complete RPMI (cRPMI), which consisted of RPMI supplemented with 10% heat-inactivated fetal bovine serum, 5% penicillin-streptomycin, 5% N-2-hydroxyethylpiperazine-N'-2-ethanesulfonic acid (HEPES), 5% GlutaMAX, and 0.5% β-mercaptoethanol.

### Adjuvants

All adjuvants were purchased from InvivoGen. These adjuvants include the following: TLR4 agonist Lipopolysaccharide from *Escherichia coli* 055:B5 (LPS) (catalog #: tlrl-pb5lps), STING agonist Bisphosphorothioate analog of 2′3′-c-di-AMP (CDN) (catalog #: tlrl-nacda2r), Vaccigrade STING agonist 2′3′-c-diAM(PS)2(Rp,Rp) endotoxin-free (CDN) (catalog #: vac-nacda2r), TLR7/8 agonist Resiquimod R848 (catalog #: tlrl-r848-5), Vaccigrade TLR7/8 agonist Resiquimod (R848) (catalog #: vac-r848), TLR4 agonist Monophosphoryl Lipid A from Salmonella minnesota R595 (MPL-A) (catalog #: tlrl-mpla), TLR9 agonist Stimulatory CpG ODN, Class C, Human/mouse (CpG) (catalog #: tlrl-m362-1), Rig-like agonist 5′ triphosphate double-stranded RNA (RLL) (catalog #: tlrl-3prna-100), and NOD1 agonist L-Ala-gamma-D-Glu-mDAP (NLL) (catalog #: tlrl-tdap).

### Antigens

Endofit Ovalbumin (OVA) (catalog #: vac-pova) was purchased from InvivoGen. GVAX is a lethally irradiated (100 Gy) tumor cell (MOC1 or B16 m-OVA), and B7H8 is a GM-CSF–secreting B16 melanoma cell line.

### Vaccine formulations

GVAX was formulated from irradiated 10^6^ B16 m-OVA or MOC1 tumor lines with 10^5^ B7H8. Each peptide vaccine contained 20 µg of endotoxin-free ovalbumin (OVA). STING ligand, CDN, formulations consisted of 0.0012–50 µg of ML–RR-S2–CDA. Resiquimod (R848; a TLR7/8 adjuvant)–based formulations are composed of 0.0012 to 50 µg of R848.

### Antibodies and flow cytometry

The anti-mouse antibodies specific for FITC CD11c (clone HL3) (catalog #: 553801), BV650 CD80 (clone 16-10A1) (catalog #: 564158), APC CD86 (clone GL1) (catalog #: 558703), BV786 CD8a (clone 53-6.7) (catalog #: 563332), BV421 MHCI (clone AF6-88.5), BV650 TNF (clone MP6-XT22) (catalog #: 563943), BV786 MHCII (clone M5/114.15.2) (catalog #: 557000), PE-594 F4/80 (clone T45-2342) (catalog #: 565613), PE CD40 (clone 3/23) (catalog #: 553791), PE-594 CD80 (clone 16-10A1) (catalog #: 562504), BV786 CD11c (clone HL3) (catalog #: 563735), BV786 CD45 (clone 30-F11) (catalog #: 564225), PE CD11c (clone HL3) (catalog #: 553802), BV786 CD4 (clone GK1.5) (catalog #: 563331), BB515 CD45 (clone 30-F11) (catalog #: 564590), and PE CD8 (clone 53-6.7) (catalog #: 553032) were purchased from BD Biosciences. Anti-human antibodies specific for BV786 CD80 (clone L307.4) (catalog #: 564159) and APC CD86 (clone 2331 (FUN-1) (catalog #: 555660) were purchased from BD Biosciences. The anti-mouse antibodies specific for PE-594 CD40 (clone 3/23) (catalog #: 124630), FITC MHCI (clone 28-8-6) (catalog #: 114606), BV605 PD-L1 (clone 10F.9G2) (catalog #: 124321), BV421 MHCII (clone M5/114.15.2) (catalog #: 107632), Alexa Fluor 700 CD11b (clone M1/70) (catalog #: 101222), Zombie aqua live/dead BV510 (catalog #: 423102), BV421 Ly6c (clone HK1.4) (catalog #: 128032), BV605 Ly6g (clone 1A8) (catalog: 127639), Alexa Fluor 488 CD45 (clone FA-11) (catalog #: 137012), APC MCHII (clone M5/114.15.2) (catalog #: 107614), BV421 CD69 (clone H1.2F3) (catalog #: 562920), BV605 CD103 (clone 2E7) (catalog #:121433), APC Nkp46 (clone 29A1.4) (catalog #: 137608), APC CD45.1 (clone A20) (catalog #: 110714), and PE-594 PD-1 (clone 29F.1A12) (catalog #: 135228) were purchased from BioLegend. Flow cytometry was performed using BD FACS Celesta Flow Cytometer. Analysis was done using FlowJo software (FlowJo LLC).

### Murine bone marrow–derived dendritic cell and macrophage generation

Briefly, bone marrow from the leg of C57BL/6 mice was plated on day 0 into 250- or 500-ml tissue culture flasks (Corning, Corning, NY) at 10^5^ cells/ml. These extracted bone marrow cells were plated in DC medium, which consisted of cRPMI supplemented with GM-CSF (20 ng/ml; BioLegend) (catalog #: 576308). On day 3, the same volume of DC medium was added to the flask. On day 6, half of the non-adherent cells were spun down and added back to the flask with an equal amount of DC media. The non-adherent cells were harvested on day 8 or 9 for mBMDC. For mBMDM, the adherent cells were harvested.

### *In vitro* mBMDC activation

On day 8, harvested mBMDCs were stimulated and cultured with monesin (for cytokines only) (catalog #: 554724), LPS (1 µg/ml), CDN (0.002 to 50 µg/ml), R848 (0.00128 to 20 µg/ml), MPL-A (0.00128 to 20 µg/ml), CPG-ODN (0.002 to 20 µg/ml), RLL (0.002 to 20 µg/ml), or NLL (0.002 to 20 µg/ml) for 24 h for surface markers and 4 h for intracellular cytokines in a 96-well plate. DCs were stained for anti-mouse CD11c, CD86, CD80, CD40, MHCII, MHCI, and TNF. Gated DC population (CD11c+) was probed for surface markers (MHCI, MHCII, CD86, CD80, and CD40) and intracellular cytokine analysis (TNFa) after permeabilization by gMFI. No data points were excluded.

### *In vitro* mBMDM and THP-1 activation

For 24 h, harvested BMDMs were stimulated and cultured on day 8 with CDN (0.0000156 to 50 µg/ml) and R848 (0.0000156 to 50 µg/ml) for the dose-response in a 96- well plate. The saturating CDN dose was 10 µg/ml, and the saturating R848 dose was 1 µg/ml. The Max-dose combines the saturating amounts of CDN (10 µg/ml) plus R848 (1 µg/ml), and the MuSyC-dose is saturating dose of CDN (10 µg/ml) plus 1/10th saturating dose of R848 (0.1 µg/ml). The mBMDMs were stained for anti-mouse F4/80, CD86, CD80, CD40, and MHCII. Gated mBMDM population (F4/80+) was measured for surface markers (MHCII, CD86, CD80, and CD40) by gMFI. The THP-1 cells were stimulated with CDN (0.78125 to 50 µg/ml) and R848 (0.78125 to 50 µg/ml) for the single-agent dose responses. The saturating CDN dose was 25 µg/ml, and the saturating R848 dose was 25 µg/ml. The Max-dose was CDN of 25 µg/ml plus R848 of 25 µg/ml, and the MuSyC-dose was CDN of 25 µg/ml plus R848 of 2.5 µg/ml. The THP-1 cells were stained for anti-mouse CD86 and CD80 and measured by gMFI. No data points were excluded.

### *In vivo* APC activation

Six- to 12-week-old female C57BL/6 were subcutaneously injected with 20 µg of Endofit ovalbumin (OVA) administered with increasing doses (0.0012 to 50 µg) of vaccigrade CDN (STING) or R848 (TLR7/8). Twenty-four hours later, the draining lymph nodes were extracted and stained for activation markers (MHCI, MHCII, CD40, CD86, and CD80). The *in vivo* doses of 20 and 2 µg were selected for CDN and R848, respectively, based on single-agent dose responses. These doses were used for the remaining *in vivo* studies. Next, the same experiment was performed, comparing the Max-dose (20 µg of CDN and 2 µg of R848) to MuSyC-dose (20 µg of CDN and 0.2 µg of R848). No data points were excluded.

### *In vivo* cytotoxic T-Cell killing assay

Six- to 12-week-old female C57BL/6 mice were vaccinated with PBS alone (n = 3), 20 µg of Endofit ovalbumin (OVA) alone (n = 3), or OVA administered with 20 µg of CDN (n = 3), 2 µg of R848 (n = 3), Max-dose (2 µg of R848 and 20 µg of CDN) (n = 4), and MuSyC-dose (0.2 and 20 µg of CDN) (n = 4) (n = 20 total). Seven days post-vaccination, splenocytes were extracted from non-vaccinated mice. Half of the splenocytes were given a high dose of 5 µM CFSE with SINFEKL peptide (1 µg/ml; specific splenocytes), and the other half was given a low dose of 0.5 µM CFSE with no SINFEKL peptide (non-specific splenocytes). The splenocytes were mixed 1:1, and then 5 × 10^6^ cells were injected intravenously into the vaccinated mice. Sixteen hours later, mice were euthanized, and spleens were extracted. Splenocytes were analyzed by flow cytometry, and specific killing was calculated with the following equation: ratio = low peak (non-specific)/high peak (OVA-specific); specific killing = (1 − (PBS ratio average)/(exp. ratio)) * 100. No data points were excluded. No animals were excluded.

### *In vivo* T-cell proliferation

CD8+ T cells were isolated from 6- to 12-week-old female CD45.1 OT-1+ mouse spleens. The CD45.1+ OT-1+ CD8+ T cells were 5 µM CFSE stained. CFSE-stained CD8 T cells (2.5 × 10^5^) were intravenously injected into naïve WT CD45.2 C57BL/6 mice (n = 3 per treatment) (n = 18 total). Twenty-four hours later, the CD45.2 C57BL/6 mice were vaccinated with PBS alone, 20 µg of Endofit ovalbumin (OVA) alone, or OVA administered with 20 µg of vaccigrade CDN (STING), 2 µg of vaccigrade R848 (TLR7/8), Max-dose (2 µg of R848 and 20 µg of CDN), and MuSyC-dose (0.2 and 20 µg of CDN). Five days post-vaccination, mice were euthanized, and spleens were extracted. Spleens were stained for CD45.1 and CD8 for flow cytometry. Percent proliferation and percentage of CD45.1+ CD8+ of total live splenocytes were calculated. No data points were excluded. No animals were excluded.

### *In vivo* tumor studies

On the basis of priori power multiple analysis of variance (MANOVA) analysis at a 95% confidence interval, power of 80%, with an intermediate (0.50) (B16 m-OVA peptide vaccine) or large effect size (0.75) (GVAX models), the total number of mice needed to reach significance for each tumor model is as follows: 25 for the B16 m-OVA GVAX model, 26 for the MOC1 GVAX model, and 34 for the B16 m-OVA peptide model. For all vaccination models, 6- to 12-week-old female C57BL/6 mice were subcutaneously injected with the following amount of cells for different tumor models in the right flank: 10^5^ B16 m-OVA tumor cells and 2 × 10^6^ MOC1 tumor cells. Five days after tumor inoculation, mice were randomized and then injected with 100 µl of GVAX B16 m-OVA (n = 28 total mice) or GVAX MOC1 (n = 43 total mice) in PBS plus or minus vaccigrade 20 µg of CDN, 2 µg of R848, Max-dose (20 µg of CDN + 2 µg of R848) or MuSyC-dose (20 µg of CDN + 0.2 µg of R848) on the opposite flank. In addition, 100 µl of PBS alone was used as a negative control. For the peptide vaccine, 20 µg of Endofit ovalbumin (OVA) (n = 11) plus or minus vaccigrade 20 µg of CDN (n = 11), 2 µg of R848 (n = 10), Max-dose (20 µg of CDN + 2 µg of R848) (n = 11), or MuSyC-dose (20 µg of CDN + 0.2 µg of R848) (n = 11) (n = 54 mice total) on the opposite flank. Tumor measurements were initiated once palpable utilizing calipers. The following formula calculated the tumor volume: length (longer dimension) × width (shorter dimension)^2^/2. OVA-based vaccine-treated tumors were extracted and weighed 16 days post-treatment. These tumors were processed and stained for immunophenotyping *via* flow cytometry. The percentage of CD45+ and the percentage of total live were evaluated utilizing the following gating: NK cells (CD11b−Nkp46+), CD8+ T cells (CD11b−Nkp46−CD4−CD8+), CD4+ T cells (CD11b−Nkp46−CD8−CD4+), gMDSC (CD11b+MHCII−CD68−CD11c−Ly6G+), mMDSC (CD11b+MHCII−CD68−CD11c−Ly6C+), DC (CD68−CD11c+MHCII+), MACs (CD11c− CD68+), M1 MACs (CD206− MACs), and M2 MACs (CD206+ MACs). No data points were excluded. Animals were excluded if the negative control had zero tumor growth throughout the entire study for a specific model, thereby excluding other zero growth from the treatment groups in the same model. One mouse from the PBS, MuSyC-dose, and Max-dose groups from the MOC1 GVAX model fits this exclusion criterion.

### *In vivo* mouse weight analysis

Six- to 12-week-old female C57BL/6 mice (n = 5 per group) (n = 30 total) were weighed to get their initial weight, considered 100%. Next, the mice were vaccinated with PBS alone, 20 µg of Endofit ovalbumin (OVA) alone, or OVA administered with 20 µg of CDN (STING), 2 µg of R848 (TLR7/8), Max-dose (2 µg of R848 and 20 µg of CDN), and MuSyC-dose (0.2 and 20 µg of CDN). Mice were weighed every 24 h for 120 h. The percent weight change was calculated daily. No data points were excluded. No animals were excluded.

### Plasma cytokine analysis

Six- to 12-week-old mice female C57BL/6 (n = 5 per group) (n = 30 total) were vaccinated with PBS alone, 20 µg of Endofit ovalbumin (OVA) alone, or OVA administered with 20 µg of CDN (STING), 2 µg of R848 (TLR7/8), Max-dose (2 µg of R848 and 20 µg of CDN), and MuSyC-dose (0.2 and 20 µg of CDN). Six hours post-vaccination, mice were euthanized, and whole blood was drawn *via* heart puncture. Plasma was separated from the blood. The following plasma cytokines were analyzed by flow cytometry utilizing the Legendplex kit (catalog #: 740150) (lot #: B326302): IL-1α, IL1β, IL-6, IL-10, IL-12p70, IL-17A, IL-23, IL-27, CCL2 (MCP-1), IFN-β, IFN-γ, TNF-α, and GM-CSF. The BioLegend Legendplex QOGNIT software quantified the concentration for each cytokine. No data points were excluded. No animals were excluded.

### The Multidimensional Synergy of Combinations analysis

Synergy was calculated using the MuSyC algorithm as previously described using a monte carlo non-linear least squares regression [35, 36]. MuSyC distinguishes two types of drug synergy, synergistic efficacy (β) and synergistic potency (α), both relating to geometric transformations of the dose response surface. These transformations are analogous to the transformations in the one-dimensional Hill equation for potency (horizontal shift in the EC_50_) and efficacy (vertical shift in e_max_). Synergy was calculated by fitting a dose-response surface relating the observed effect (i.e., change in surface marker expression) to the concentrations of CDN and the tested adjuvant (**Figures 1**, **S3**). As the maximal effect of the drugs (e_max_) is larger than the basal effect (E0) when quantifying mBMDC activation, synergistic efficacy (beta) is defined as (e_max_ − max(E1,E2))/(max(E1,E2) − E0), where E3 is the effect observed at the maximum of both drugs, E1 is the maximum effect for drug 1 alone, and E2 is the maximum effect for drug 2 alone. No bounds were required for the non-linear regression to converge.

### Statistical analysis

Multiple comparison tests of datasets were achieved with a one-way or two-way analysis of variance (ANOVA). Error bars reflect the standard error of the mean (SEM) or the standard deviation (S.D.). Tests of significance are reported as P-values, a two-tailed distribution, and calculated at 95% confidence. All data analyses were performed using Graphpad prism. *A priori* analyses were performed utilizing the G-power 3.1.9.7 calculator.

## Data availability statement

The raw data supporting the conclusions of this article will be made available by the authors, without undue reservation.

## Ethics statement

The animal study was reviewed and approved by Institutional Animal Care and Use Committee at Vanderbilt University Medical Center (M1900004-00).

## Author contributions

Experimental design: DT, DG, MK, CM, VQ, RS, JF, YK, and JW. Performed experiments: DT, MK, DG, JF, RS, ET, BM, GR, CL, and HF. Analysis and interpretation of data: DT, MK, CM, YK, and JW. Manuscript preparation: DT, MK, CM, VQ, RS, YK, and JW. All authors contributed to the article and approved the submitted version.

## Funding

This work was supported by the Barry Baker Biorepository Fund (YK), NIH R01 CA178613 (YK), R01 DE027749 (YK), Department of Defense CDMRP Breakthrough Award (YK).

## Acknowledgments

We thank Kyle Becker for his contribution to the mouse cytokine experiment.

## Conflict of interest

DT and MK report grants from DoD USAMRAA and NIH, as well as other support from Barry Baker Biorepository Fund and James Rowen Fund during the conduct of the study. YK reports grants from NCI and NIDCR during the conduct of the study, as well as personal fees from Aduro, AstraZeneca, Sanofi, Takeda, and Mersanna outside the submitted work.

Author YK was employed by Regeneron Pharmaceuticals, Inc.

The remaining authors declare that the research was conducted in the absence of any commercial or financial relationships that could be construed as a potential conflict of interest.

## Publisher’s note

All claims expressed in this article are solely those of the authors and do not necessarily represent those of their affiliated organizations, or those of the publisher, the editors and the reviewers. Any product that may be evaluated in this article, or claim that may be made by its manufacturer, is not guaranteed or endorsed by the publisher.

## Supplementary material

Supplementary Figure 1 STING agonist is the most efficacious adjuvant for stimulating murine dendritic cells. **(A)** Gating strategy for murine bone marrow–derived dendritic cells (BMDCs) **(B)** Dose-response to identify saturating dose for CD86 signal (geometric mean fluorescence intensity, gMFI) for STING (CDN), TLRs (CpG, MPL-A, and R848), NOD (NLL), and RIG-I (RLL). Saturating doses: CDN (20µg/mL), R848 (2µg/mL), CpG (20µg/mL), MPL-A (2µg/mL), NLL (0.02µg/mL), and RLL (0.02µg/mL). 1µg/mL is the dose used for LPS. **(C)** Expression levels of MHCI, CD86, and TNF at the saturating dose for each adjuvant. All data are given in mean ± S.D. of 3 technical replicates. *P < 0.05, **P < 0.01, ***P < 0.001, ****P < 0.0001, one-way analysis of variance (ANOVA) for multiple comparisons.

Supplementary Figure 2 Both MPL-A and R848 potentiate the stimulatory effects of CDN on DCs. TNF, CD40, and MHCI expression on mouse bone marrow–derived dendritic cells (mBMDCs) after stimulation with saturating CD86 dose (**Supplementary Figure 1**) of CDN (STING) plus saturating dose of TLRs (CpG, MPL, and R848), NOD (NLL), and RIG-I (RLL) termed the Max-dose. All data are given in mean ± S.D. of 3 technical replicates. *P < 0.05, **P < 0.01, ***P < 0.001, ****P < 0.0001, one-way analysis of variance (ANOVA) for multiple comparisons (bracket) of STING alone versus the combinations and two-way ANOVA analysis of variance for comparison (non-bracket) of no CDN vs. the addition of CDN.

Supplementary Figure 3 The combination of CDN and MPL-A is synergistically potent. **(A)** Comprehensive checkerboard plate map for combining STING and TLR agonists. **(B)** MuSyC algorithm-generated drug synergy diagrams for STING and TLR4 agonists to activate mBMDCs. The y-axis is the log concentration of CDN (STING), the x-axis is the log concentration of MPL (TLR4), and the z-axis is the geometric mean fluorescence intensity (gMFI) of multiple activation markers. Points are experimentally measured conditions. The surface is the fit to the MuSyC equation, which quantifies the synergistic potency and efficacy. The solid red line is the MPL single-agent dose-response. The solid blue line is CDN single-agent dose-response. The blue dashed line is the max dose of MPL plus increasing amounts of CDN. The red dashed line represents the max dose of CDN plus increasing doses of MPL. **(C)** One-dimensional graphs displaying an open circle for the EC_50_ for the single agents and a solid black circle for the new EC_50_ in the presence of the combinatorial agent. The vertical dashed lines represent the EC_50_ of the respective curve.

Supplementary Figure 4 Dose responses for murine bone marrow-derived macrophages (mBMDM) and human monocytic cell line THP-1. **(A)** Corresponding dose-response curves for CDN (STING) and R848 (TLR7/8) for the activation of mBMDM with saturation range in the red box. 10µg/mL was chosen for CDN, and 1µg/mL was chosen for R848. **(B)** 25µg/mL was chosen for CDN, and 25µg/mL was chosen for R848 for the activation of human monocytic cell line THP-1.

Supplementary Figure 5 Gating for dendritic cells. Gating scheme for lymph node dendritic cells (CD11c+ MHCII+).

Supplementary Figure 6 CDN and combinations saturate T-cell proliferation. CFSE low percentage 5-days post-vaccination with corresponding vaccines. All data are given in mean ± S.D. *P < 0.05, **P < 0.01, ***P < 0.001, ****P < 0.0001, one-way analysis of variance (ANOVA) for multiple comparisons.

Supplementary Figure 7 Gating for tumor immunophenotypingGating scheme for immunophenotyping.
