# Supplementary figures and images for: MuSyC dosing of adjuvanted cancer vaccines optimizes antitumor responses
Source: Front Immunol. 2022 Aug 19;13:936129. doi: 10.3389/fimmu.2022.936129 (PMC9437625; doi:10.3389/fimmu.2022.936129)

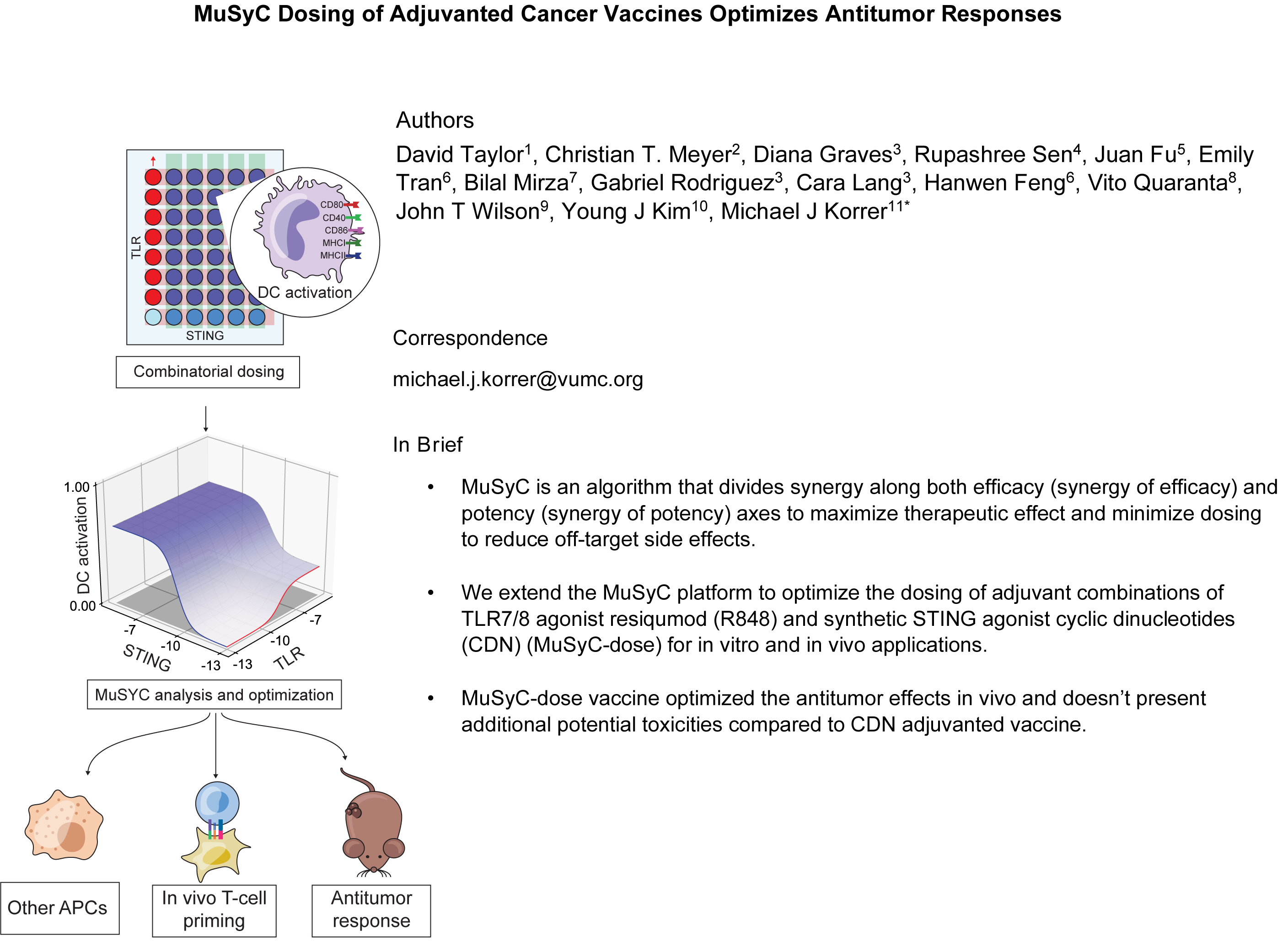

Supplement: Supplementary file 1 [file Image_8.tif]

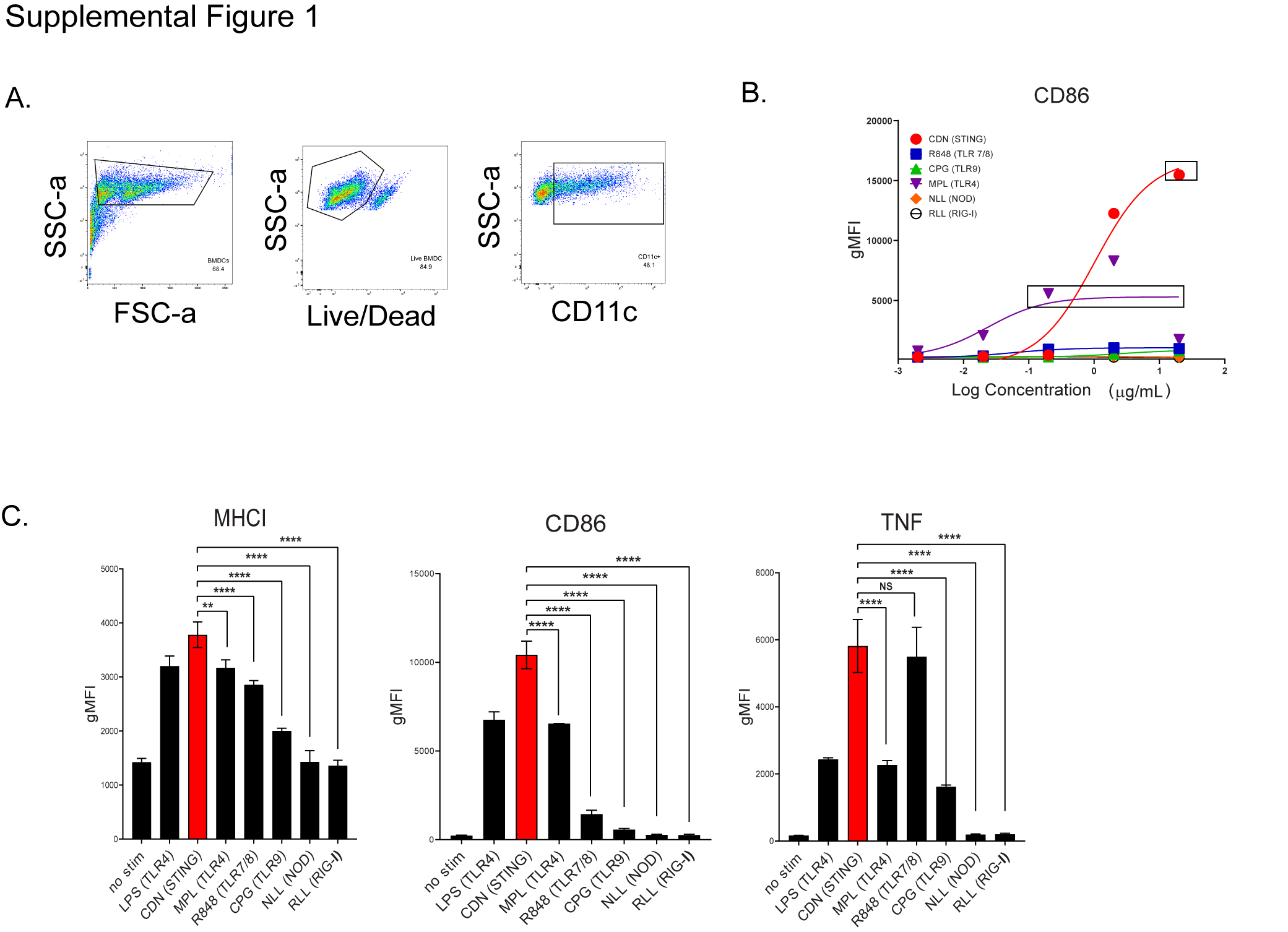

Supplement: Supplementary Figure 1 — STING agonist is the most efficacious adjuvant for stimulating murine dendritic cells. (A) Gating strategy for murine bone marrow–derived dendritic cells (BMDCs) (B) Dose-response to identify saturating dose for CD86 signal (geometric mean fluorescence intensity, gMFI) for STING (CDN), TLRs (CpG, MPL-A, and R848), NOD (NLL), and RIG-I (RLL). Saturating doses: CDN (20µg/mL), R848 (2µg/mL), CpG (20µg/mL), MPL-A (2µg/mL), NLL (0.02µg/mL), and RLL (0.02µg/mL). 1µg/mL is the dose used for LPS. (C) Expression levels of MHCI, CD86, and TNF at the saturating dose for each adjuvant. All data are given in mean ± S.D. of 3 technical replicates. *P < 0.05, **P < 0.01, ***P < 0.001, ****P < 0.0001, one-way analysis of variance (ANOVA) for multiple comparisons. [file Image_1.tif]

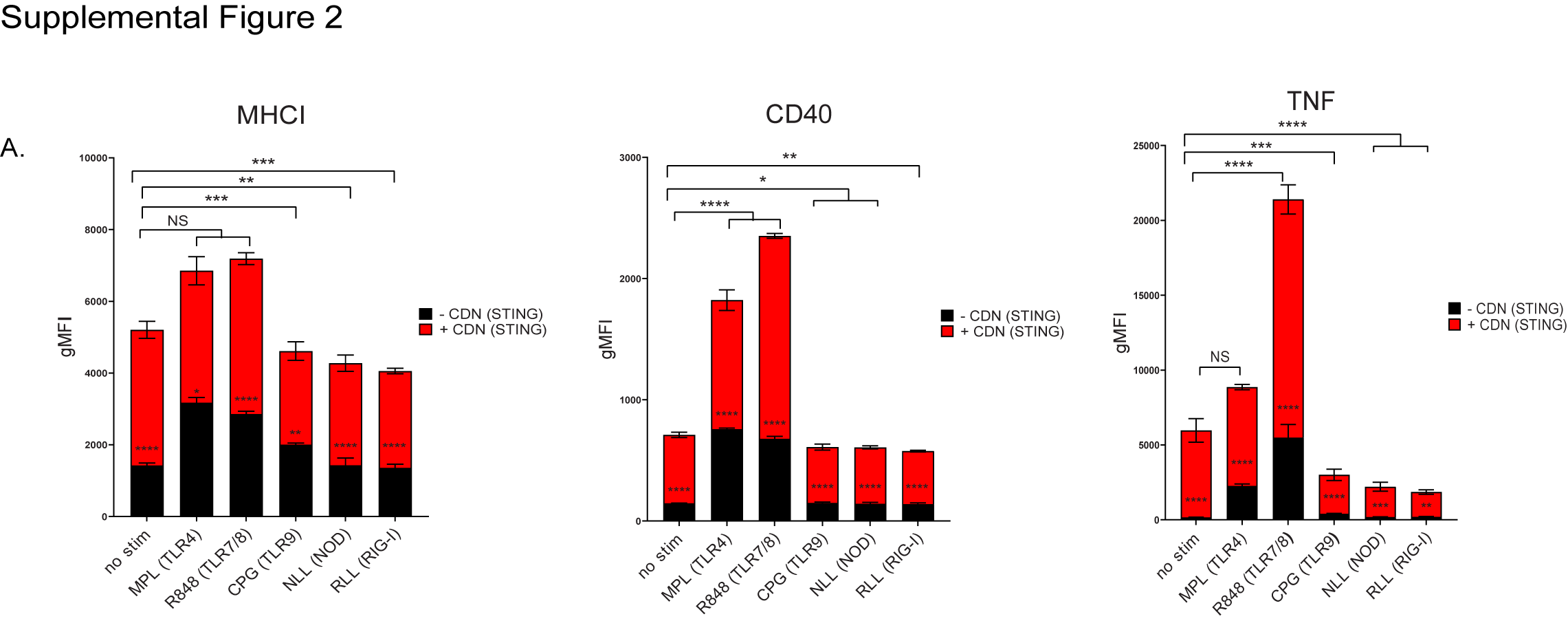

Supplement: Supplementary Figure 2 — Both MPL-A and R848 potentiate the stimulatory effects of CDN on DCs. TNF, CD40, and MHCI expression on mouse bone marrow–derived dendritic cells (mBMDCs) after stimulation with saturating CD86 dose ( Supplementary Figure 1 ) of CDN (STING) plus saturating dose of TLRs (CpG, MPL, and R848), NOD (NLL), and RIG-I (RLL) termed the Max-dose. All data are given in mean ± S.D. of 3 technical replicates. *P < 0.05, **P < 0.01, ***P < 0.001, ****P < 0.0001, one-way analysis of variance (ANOVA) for multiple comparisons (bracket) of STING alone versus the combinations and two-way ANOVA analysis of variance for comparison (non-bracket) of no CDN vs. the addition of CDN. [file Image_2.tif]

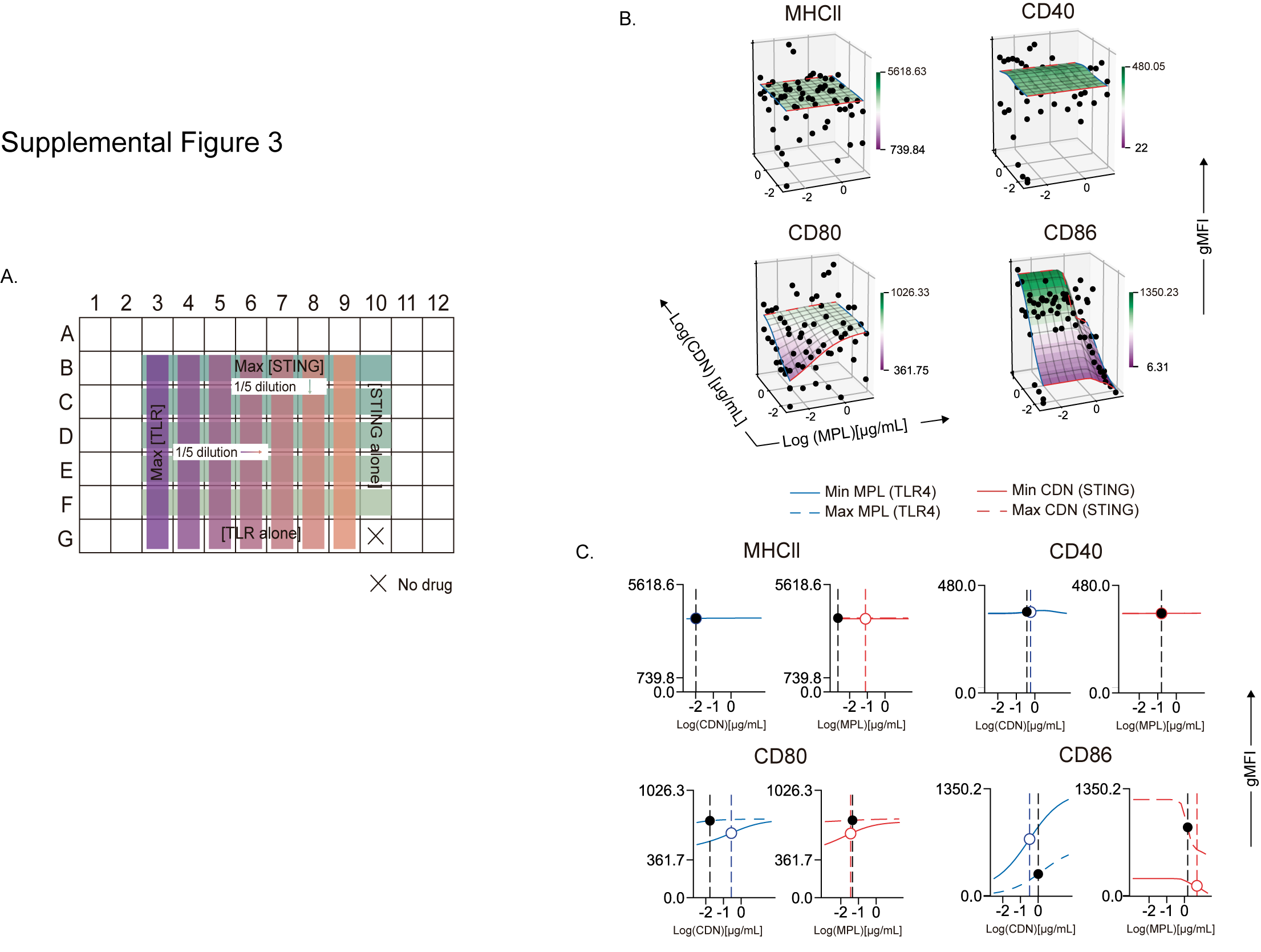

Supplement: Supplementary Figure 3 — The combination of CDN and MPL-A is synergistically potent. (A) Comprehensive checkerboard plate map for combining STING and TLR agonists. (B) MuSyC algorithm-generated drug synergy diagrams for STING and TLR4 agonists to activate mBMDCs. The y-axis is the log concentration of CDN (STING), the x-axis is the log concentration of MPL (TLR4), and the z-axis is the geometric mean fluorescence intensity (gMFI) of multiple activation markers. Points are experimentally measured conditions. The surface is the fit to the MuSyC equation, which quantifies the synergistic potency and efficacy. The solid red line is the MPL single-agent dose-response. The solid blue line is CDN single-agent dose-response. The blue dashed line is the max dose of MPL plus increasing amounts of CDN. The red dashed line represents the max dose of CDN plus increasing doses of MPL. (C) One-dimensional graphs displaying an open circle for the EC50 for the single agents and a solid black circle for the new EC50 in the presence of the combinatorial agent. The vertical dashed lines represent the EC50 of the respective curve. [file Image_3.tif]

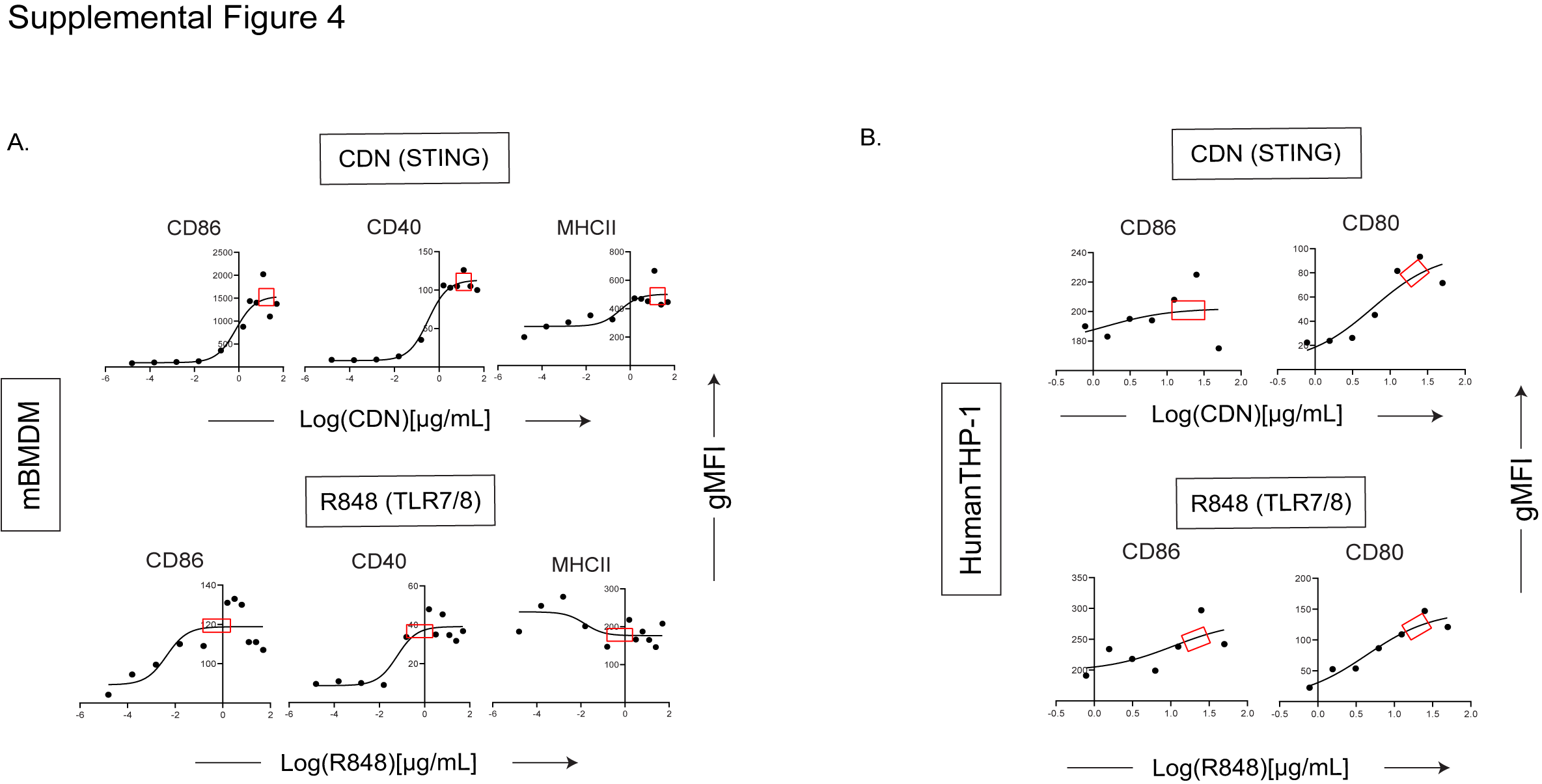

Supplement: Supplementary Figure 4 — Dose responses for murine bone marrow-derived macrophages (mBMDM) and human monocytic cell line THP-1. (A) Corresponding dose-response curves for CDN (STING) and R848 (TLR7/8) for the activation of mBMDM with saturation range in the red box. 10µg/mL was chosen for CDN, and 1µg/mL was chosen for R848. (B) 25µg/mL was chosen for CDN, and 25µg/mL was chosen for R848 for the activation of human monocytic cell line THP-1. [file Image_4.tif]

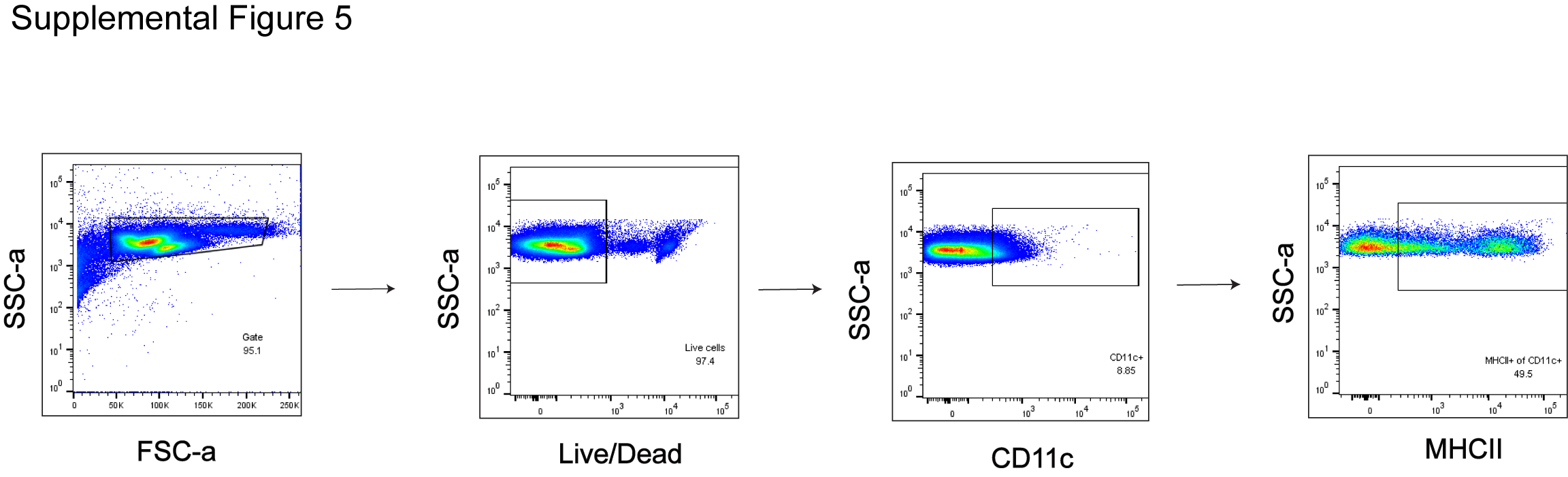

Supplement: Supplementary Figure 5 — Gating for dendritic cells. Gating scheme for lymph node dendritic cells (CD11c+ MHCII+). [file Image_5.tif]

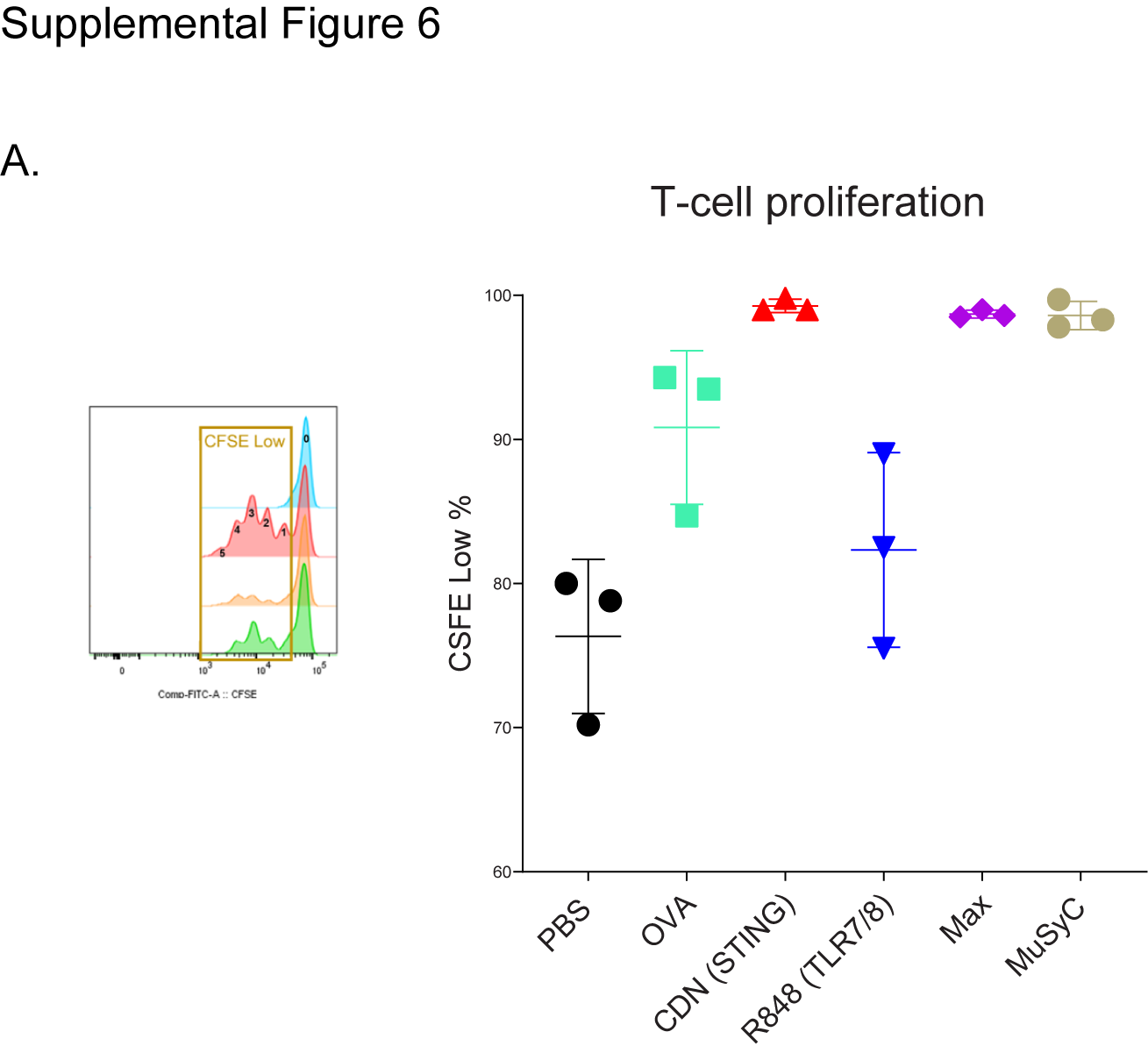

Supplement: Supplementary Figure 6 — CDN and combinations saturate T-cell proliferation. CFSE low percentage 5-days post-vaccination with corresponding vaccines. All data are given in mean ± S.D. *P < 0.05, **P < 0.01, ***P < 0.001, ****P < 0.0001, one-way analysis of variance (ANOVA) for multiple comparisons. [file Image_6.tif]

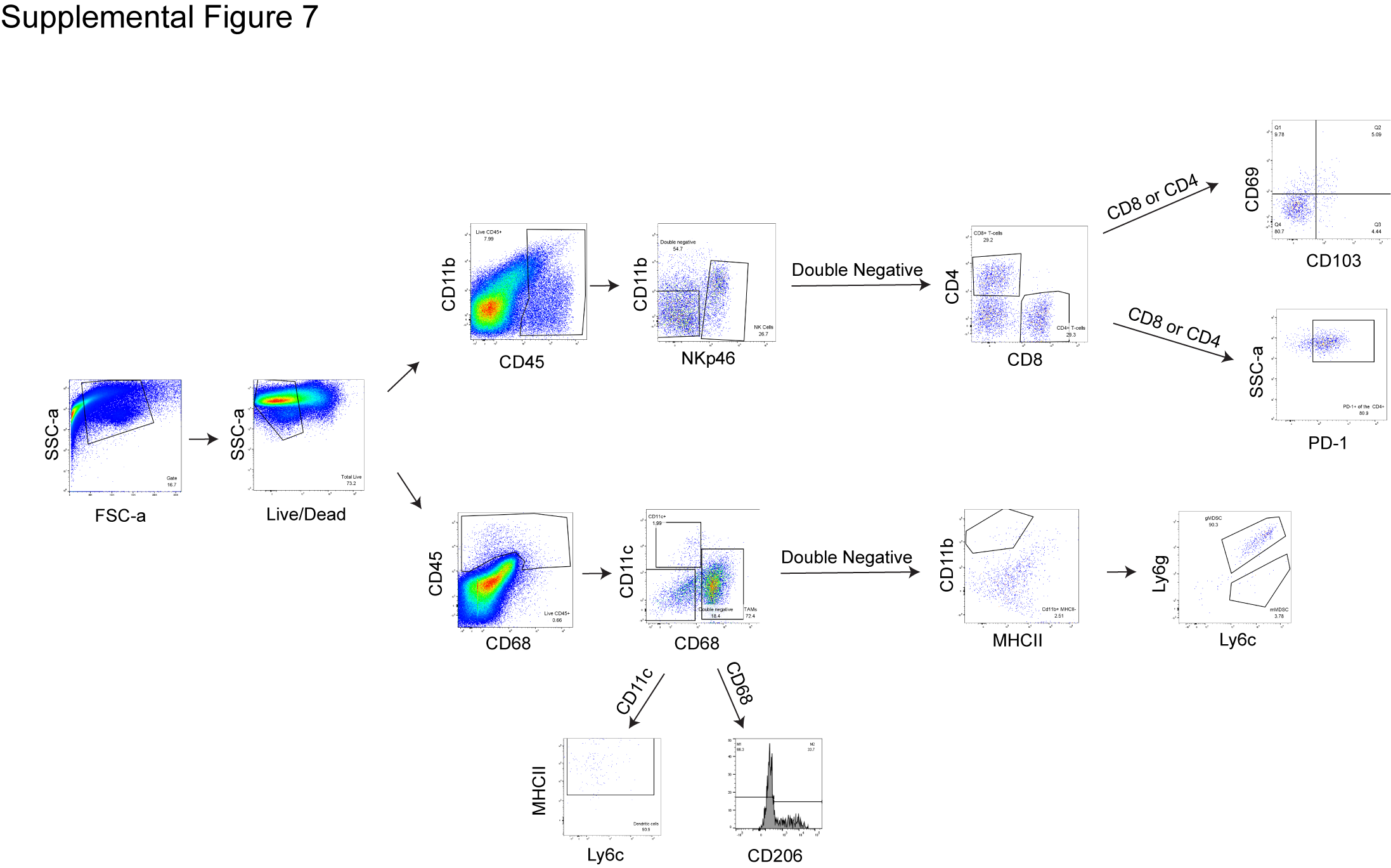

Supplement: Supplementary Figure 7 — Gating for tumor immunophenotypingGating scheme for immunophenotyping. [file Image_7.tif]
